# Laser‐Engraved Micro‐Patterned rGO/WPU Composite Structures for Aircraft Applications: Synchronizing Electrothermal De‐Icing and Broadband Microwave Transparency

**DOI:** 10.1002/advs.202512521

**Published:** 2025-09-08

**Authors:** Xu Fu, Yizhou Shen, Lingfeng Zhao, Weixin Zhu, Weilan Liu, Chenliang Li, Yuebin Lin, Zongsheng Ye, Chengfeng Shen

**Affiliations:** ^1^ College of Materials Science and Technology Nanjing University of Aeronautics and Astronautics Nanjing 211100 P. R. China; ^2^ State Key Laboratory of Mechanics and Control for Aerospace Structures Nanjing University of Aeronautics and Astronautics Nanjing 210016 P. R. China; ^3^ Institute of Advanced Materials Nanjing Tech University 30 Puzhu South Rd. Nanjing 210009 P. R. China; ^4^ Faculty of Mechanical and Material Engineering Huaiyin Institute of Technology Huai'an 223003 P. R. China; ^5^ ZhongXing Energy Enquipment Co,Ltd. Nantong 226100 P. R. China

**Keywords:** electrothermal properties, frequency selective surface, incoming flow environment, patterned design, wave‐transparent properties

## Abstract

Aircraft confronting harsh meteorological conditions and radar detection environments during high‐altitude flights face significant risks, which can threaten flight safety. This study designs and fabricates a novel Jerusalem cross‐inspired Frequency Selective Surface (FSS). Initially, rGO powder with an optimized reduction degree is synthesized as the conductive filler. Based on this, the rGO/WPU electrothermal coating is prepared using the spraying method, demonstrating excellent electrical conductivity and electrothermal performance. Subsequently, a New Jerusalem Pattern Structure (NJPS) is engineered on the coating surface. This patterned design notably improved microwave transmittance. Parameter optimization ensured superior wave transmission performance, with the underlying mechanism explained through antisymmetric surface current analysis. Laser‐engraved NJPS samples are tested under both room‐temperature and low‐temperature conditions, confirming dual functionality: effective Joule heating and stable broadband transmission. Importantly, icing wind tunnel tests under simulated flight conditions verified the de‐icing capability within 216s via interfacial water‐film lubrication. This patterning strategy establishes a viable methodology for reconciling electrothermal de‐icing and electromagnetic transparency on aircraft surfaces.

## Graphical abstract

While conventional electrothermal materials tend to reflect electromagnetic waves during de‐icing. Inspired by the frequency‐selective surface theory, this paper designs NJPS for rGO/WPU electrothermal coatings based on the traditional Jerusalem structure. NJPS can ensure excellent electric deicing capacity in low‐temperature flow environments and has outstanding electromagnetic wave transmission performance, offering a balanced solution for stealth and thermal performance.

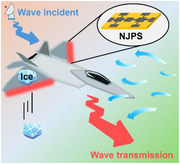

## 1 Introduction

Rapid advances in modern aviation technology make the reliability and versatility of aircraft in extreme environments an increasingly urgent requirement. Supercooled liquid droplets freeze upon impact on their windward surfaces when aircraft traverse supercooled cloud layers.^[^ ^1^, ^2^, ^3^, ^4^, ^5^, ^6^ ^]^ The accretion of ice can significantly alter the flow field near the aircraft surface. This results in reduced lifts, increased drag, degradation of flight stability, and compromised controllability. These adverse effects can lead to safety incidents.^[^ ^7^, ^8^, ^9^, ^10^ ^]^ Concurrently, advancements in radar technology are deteriorating the aircraft's operational environment.^[^ ^11^ ^]^ Furthermore, electromagnetic wave transmittance, a prerequisite for stealth capability, critically determines overall stealth performance. Both factors—the hazardous icing effects and the demands of operating in sophisticated radar environments while maintaining stealth—pose significant challenges to aircraft flight safety and combat survivability.^[^ ^12^, ^13^, ^14^, ^15^, ^16^, ^17^, ^18^, ^19^ ^]^

In recent years, researchers have developed various anti/de‐icing technologies to address the problem of aircraft surface icing. Such as superhydrophobic anti‐icing,^[^ ^20^, ^21^, ^22^ ^]^ electrothermal de‐icing,^[^ ^23^, ^24^ ^]^ and vibrational de‐icing.^[^ ^25^, ^26^, ^27^ ^]^ Among these, electrothermal de‐icing has been extensively studied due to its high de‐icing efficiency and operational stability. Conventional electrothermal de‐icing systems employ metallic materials. However, electromagnetic waves experience significant reflection when passing through metallic films, preventing the simultaneous achievement of electrothermal de‐icing and electromagnetic wave transmission. The skin depth (*δ*), which governs the penetration of electromagnetic waves into a conductive surface, can be calculated as follows^[^ ^28^ ^]^:

(1) $$\delta =\sqrt{\frac{\rho }{\pi f\mu }}=\sqrt{\frac{1}{\pi f\mu \sigma }}$$

where *f* denotes the frequency of the electromagnetic wave, *μ* represents the magnetic permeability, *ρ* is the electrical resistivity, and *σ* is the electrical conductivity (where *σ* = 1/*ρ*).

This equation demonstrates that electromagnetic waves cannot effectively penetrate the material beyond a depth of *δ*. Due to the very high electrical conductivity of metallic heating elements, a large portion of incident electromagnetic waves is reflected when they strike the surface. In contrast, carbon‐based electrothermal thin films possess microwave‐transparent properties, indicating that carbon‐based electrothermal films are a promising approach for simultaneously enabling electromagnetic wave transmission and electrical heating capabilities.^[^ ^29^ ^]^

In recent years, research on the integration of electrothermal de‐icing and electromagnetic wave transmission functions has been increasingly reported.^[^ ^30^, ^31^ ^]^ Hong^[^ ^32^ ^]^ developed a highly radio frequency transparent electrothermal film based on carbon nanotubes (CNTs) using a simple spin‐coating method. Although increasing CNT content improved electrothermal performance, the accompanying reduction in film resistivity caused a decline in electromagnetic wave transmission. Consequently, a CNT film with optimized properties was produced, showing high RF transmittance (>80% within a specific frequency range) and effective heating performance (160 °C at 80 V). However, this method, which depends on modifying the electrothermal film material itself to improve its electromagnetic transmission capabilities, only worked within limited frequency bands and could not meet the needs for broadband wave transmission.

Recently, significant research has focused on Frequency Selective Surfaces (FSS).^[^ ^33^, ^34^, ^35^, ^36^, ^37^, ^38^, ^39^, ^40^ ^]^ FSS technology involves the periodic arrangement of conductive patches or apertures on a conductive surface or the regular distribution of gaps across a dielectric surface. This technique allows electromagnetic waves of certain frequencies to pass through or be blocked, effectively acting as a spatial filter.^[^ ^41^, ^42^ ^]^ Chen^[^ ^29^ ^]^ designed and fabricated a patterned electrothermal composite film (PEF) that combines high microwave transmittance with anti/de‐icing capabilities. This PEF achieved transmittance above 80% over an ultra‐wide frequency range of 2–18 GHz. Additionally, the film showed anti‐icing performance at a heating power density of 0.4 W cm^−^^2^. Similarly, Liu^[^ ^43^ ^]^ proposed a gridded CNT electrothermal film that enables efficient surface heating and high transmittance. Under vertically incident transverse magnetic (TM) polarized waves, this structure achieved transmittance over 50% across the 2–18 GHz band and more than 80% within the 2–8.88 GHz sub‐band. The structure also demonstrated excellent heating efficiency and a fast electrothermal response time. At a power density of 4629.6 W m^−^^2^ and an ambient temperature of −20 °C, the CNT film successfully removed surface ice within 110 s. In summary, patterned designs based on FSS show great potential for enabling both electrothermal de‐icing and electromagnetic wave transmission. However, a key challenge remains: the main icing area on aircraft wings is the airfoil leading edge, and traditional low‐temperature environmental tests cannot fully replicate the dynamic airflow conditions experienced during actual flight.

In this work, a Novel Jerusalem Patterned Structure (NJPS) was fabricated to achieve integrated electrothermal de‐icing and electromagnetic wave transmission. The rGO/WPU electrothermal film was designed, manufactured, and tested for its electrical properties and evaluated for its electrothermal performance. Inspired by Frequency Selective Surface (FSS) principles, the NJPS was designed based on a conventional Jerusalem cross unit cell. The geometric parameters were simulated and optimized to ensure excellent wave transmission performance. At the same time, the wave transmission characteristics of the NJPS under varying oblique incidence angles were systematically investigated. The NJPS was then fabricated using laser engraving techniques. Its electrothermal performance and broadband electromagnetic wave transmission properties were evaluated under diverse environmental conditions. Importantly, the de‐icing behavior of the NJPS under realistic airflow conditions was validated through an icing wind tunnel simulation. This strategy effectively addresses the inherent conflict between achieving high‐efficiency electrothermal de‐icing and broadband electromagnetic wave transmission in thin‐film systems. Overall, this approach provides a practical design strategy for future integrated electrothermal/transparent‐wave structures.

## 2 Experimental Section

### 2.1 Material

H_2_SO_4_ (98%, mass fraction, the same below), H_3_PO_4_ (85%), KMnO_4_ (99.5%), HCl (36%–38%), H_2_O_2_ (30%) were purchased from Sinopharm Chemical Reagent Co. Ltd.; Graphite (99.8%, 32 mesh) was purchased from Qingdao Tengshengda Carbon Machinery Co. Ltd. WPU with a solid content of 60 % and sodium dodecyl sulfate (SDS) was obtained from Macklin Biochemical Technology Co. Ltd. Ethanol solvent was purchased from Aladdin Chemical Reagent Co. Polyimide film (PI), size 9*9 cm was used as the substrate for electrical and thermal performance testing, 18 cm*18 cm was used as the substrate for free‐space testing of wave‐transparent performance, the thickness of which was 0.1 mm, all were provided as the substrate by Nanjing Suqida Co. The deionized water is homemade in the laboratory.

### 2.2 Preparation of NJPS

**Figure 1:**
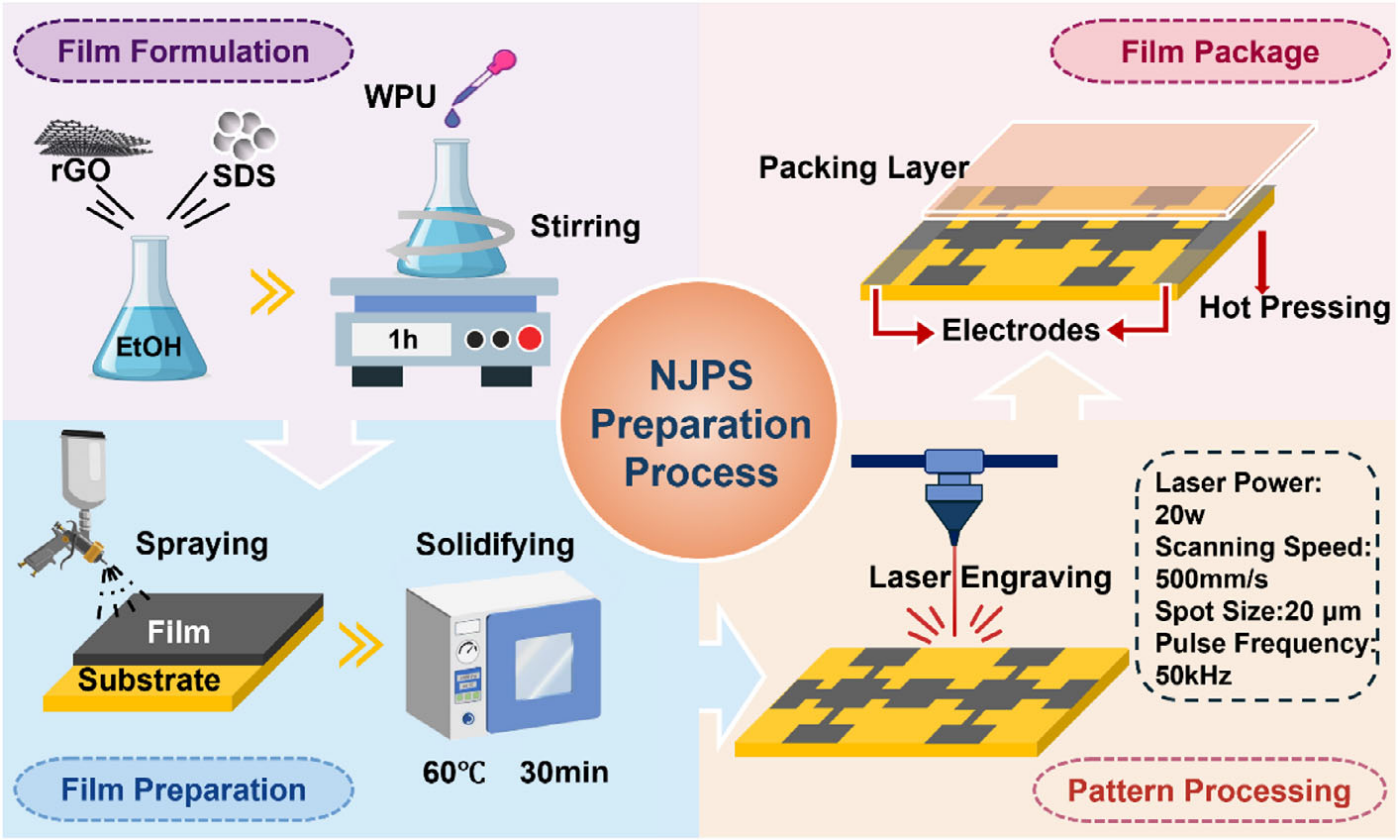
Fabrication process of NJPS.

rGO powder was prepared by the Hummers method at a high temperature of 800°C. The process of preparing for NJPS is shown in **Figure** **1**. Firstly, rGO, acting as a conductive filler, and SDS, accounting for 1 wt.% rGO by weight, were added to an ethanol solution and mixed under ultrasonic vibration for 15 min. It is worth noting that the mass ratio of ethanol to rGO was 40:1 to ensure the mixed solution had sufficient fluidity for spraying. Additionally, 2 g of WPU was added to the mixture, and the mixture was stirred magnetically at room temperature for 1 h to obtain a homogeneous conductive solution. The conductive solution was sprayed onto the substrate and cured at 60 °C for 60 min to obtain the rGO electrothermal coating. Novel Jerusalem Patterned Structures (NJPS) were created based on the obtained rGO electrothermal coatings. The pure electrothermal coatings were then engraved using an infrared laser to form a specific pattern. Two different laser passes with varying power levels are required to remove the residual powder from the surface and achieve the NJPS. Subsequently, the copper foil electrode sheets and the upper encapsulation layer are hot‐pressed onto the NJPS surface by a hot‐pressing process.

### 2.3 Characterization

The physical phase was analyzed using an XRD instrument (D8 Advance type) with a Cu Kα target (λ = 0.15406 nm). The chemical composition was analyzed using XPS (Es calab 250Xi type). The chemical structure of the samples was analyzed using Raman spectroscopy (inVia type) with an excitation wavelength of 532 nm and a spectral range of 500–3500 cm^−1^. The surface morphology of the electrothermal coatings was examined using a scanning electron microscope (SEM, Japan Hitachi Regulus 8100). The conductivity of the electrothermal coatings was measured with a multimeter (Agilent Technologies, Santa Clara, CA, USA). A DC power supply (YGX300V‐3A) was applied to the electrothermal coating and the NJPS to evaluate electrothermal behavior. Subsequently, the de‐icing performance of the NJPS was tested using an icing wind tunnel environment. Additionally, a vector network analyzer (Agilent N5244A) was used to evaluate the wave transmission performance of the NJPS by measuring the transmission coefficient of the sample.

### 2.4 Simulation

Electromagnetic Wave Simulation Technology (CST) Microwave Studio was used to simulate the transmission coefficients and transmittance, as well as to analyze the distribution of the electromagnetic field in the structure at different frequency bands. To simplify the prepared NJPS, resistive sheets were chosen instead of electrothermal films for designing the periodic cell structure. The boundary conditions are set as unit cells in the x‐y direction, open (add space) in the Z_min_ and Z_max_ directions. The Floquet port excitation port is set at the top of the air cavity. The incident electromagnetic wave is vertically incident along the ‐Z direction, in the mode of a transverse magnetic wave (TM) or a transverse electric wave (TE). The frequency range of the solutions is 2 GHz‐18 GHz. The frequency range is 2–18 GHz, with a step size of 0.1 GHz. The S‐parameters are simulated to obtain the transmittance.

## 3 Results and Discussion

### 3.1 Electrical and Electrothermal Properties of rGO/WPU Coatings

**Figure 2:**
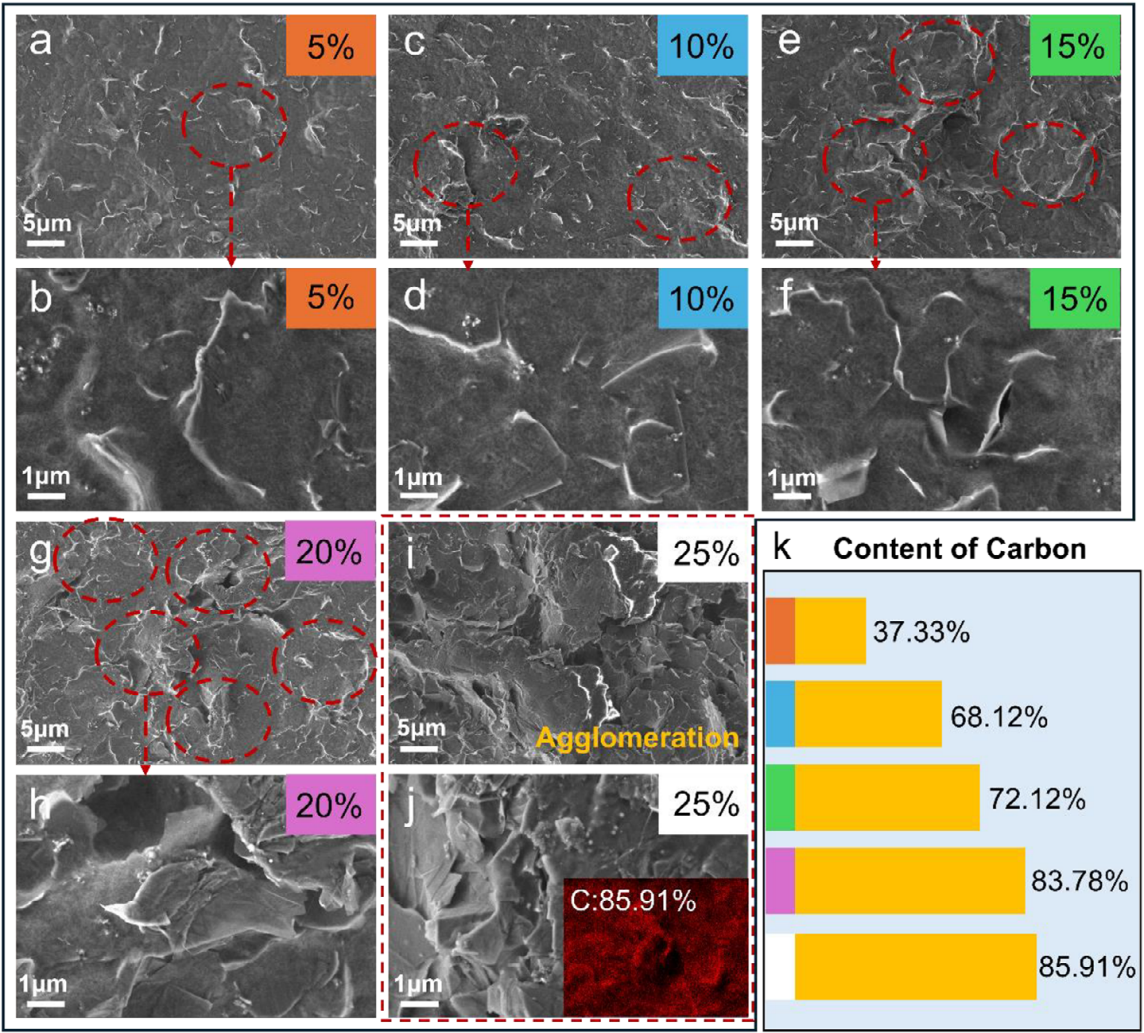
a–j) Surface microscopic morphology of electrothermal coatings with different rGO contents; k) The carbon content on the surface of rGO/WPU electrothermal coating.

First, the prepared rGO powder was tested, and the results are shown in Figure S1 (Supporting Information), demonstrating its excellent reduction degree. Then, the rGO/WPU electrothermal coatings were made using a spray‐coating method. The surface morphology of coatings with different rGO contents (5–25 wt.%) was first examined, as shown in **Figure** **2**a–j. At a low rGO loading of 5 wt.%, a conductive network forms; however, the distribution of rGO sheets on the surface appears sparse. As the rGO content increases, more flake‐like rGO sheets become evenly distributed across the coating surface, creating a clearly visible, denser, and more continuous conductive network. However, at the highest rGO content of 25 wt.%, noticeable agglomeration is observed within the coating microstructure. This agglomeration results from the inherent dispersion instability of rGO, which hampers its complete distribution within the solution and eventually causes the aggregation of conductive filler on the coating surface. This aggregation reduces the uniformity of the conductive pathways within the coating. Meanwhile, as shown in Figure 2k, the carbon content in the coating increases with the rGO content, indicating that rGO contributes to the carbon in the coating.

**Figure 3:**
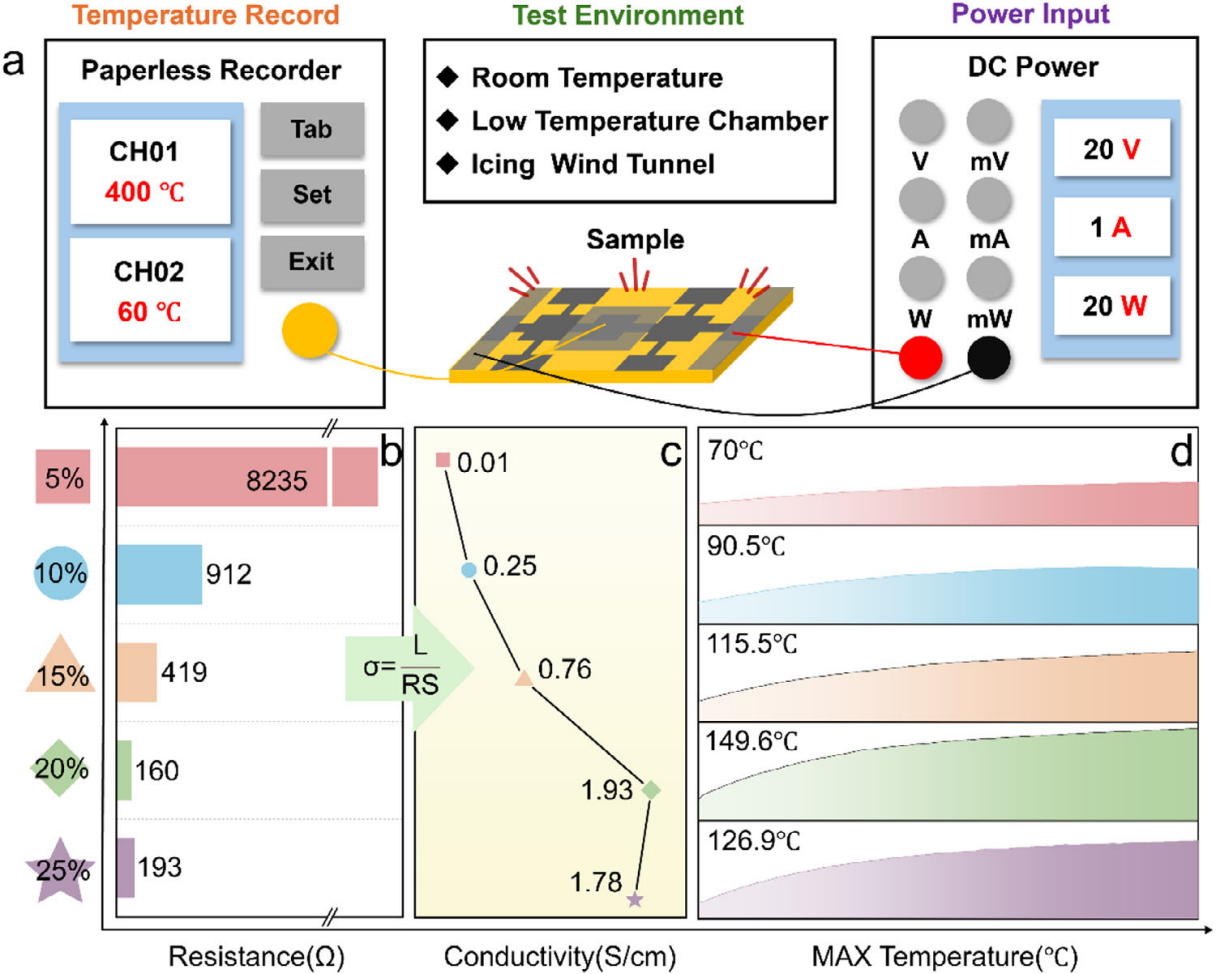
a) Schematic diagram of electric heating performance test; Test results of coatings with different contents of rGO: b) Resistance; c) Conductivity; d) Electrothermal properties at room temperature.

The electrical resistance of coatings with different rGO loadings was then measured (**Figure** **3**a). The coating with only 5 wt.% rGO shows a high resistance of 8235 Ω. As the rGO mass fraction increases, the resistance drops significantly, reaching a minimum of 160 Ω at 20 wt.% rGO. Interestingly, increasing the rGO content further to 25 wt.% causes an unusual increase in resistance to 193 Ω. The electrical conductivity of the coatings was then calculated from their measured resistance and thickness. Conductivity is derived from the electrical resistivity, which is calculated using Equation (2):

(2) $$\rho =\frac{RS}{L}$$

where:*ρ* is the electrical resistivity, *R* is the measured resistance, *S* is the cross‐sectional area of the coating, and *L* is the distance between the electrodes. The conductivity (*σ*) is the reciprocal of resistivity:

(3) $$\sigma =\frac{1}{\rho }=\frac{L}{RS}$$

The calculated electrical conductivity values are shown in Figure 3b. The conductivity trend is inversely related to resistance. At the lowest rGO loading of 5 wt.%, the coating has a conductivity of 0.01 S cm^−1^. The conductivity increases significantly with increasing rGO mass fraction, reaching a maximum value of 1.93 S cm^−1^ at 20 wt.% rGO. This is about a 200‐fold increase in conductivity, despite only a four‐fold increase in rGO mass. However, increasing rGO content to 25 wt.% leads to a decrease in conductivity. This decrease is attributed to the agglomeration of conductive filler at high rGO loadings, which disrupts the uniform conductive pathways in the coating. Subsequently, a constant voltage was applied to coatings with different rGO loadings at room temperature for 120 s to evaluate the electrothermal performance. The temperature rise profiles, shown in Figure 3c, reveal a similar heating pattern: a rapid initial temperature increase followed by stabilization at a steady state.^[^ ^44^ ^]^ Notably, the coating with 20 wt.% rGO reaches the highest steady temperature of 149.6 °C within the 120s heating period. This superior heating performance is directly linked to its highest electrical conductivity, as previously established. In summary, the rGO/WPU electrothermal coatings provide quick and efficient heating at room temperature, confirming their feasibility for applications such as de‐icing.

### 3.2 Novel Jerusalem Patterned Structure (NJPS) on rGO/WPU Coatings

**Figure 4:**
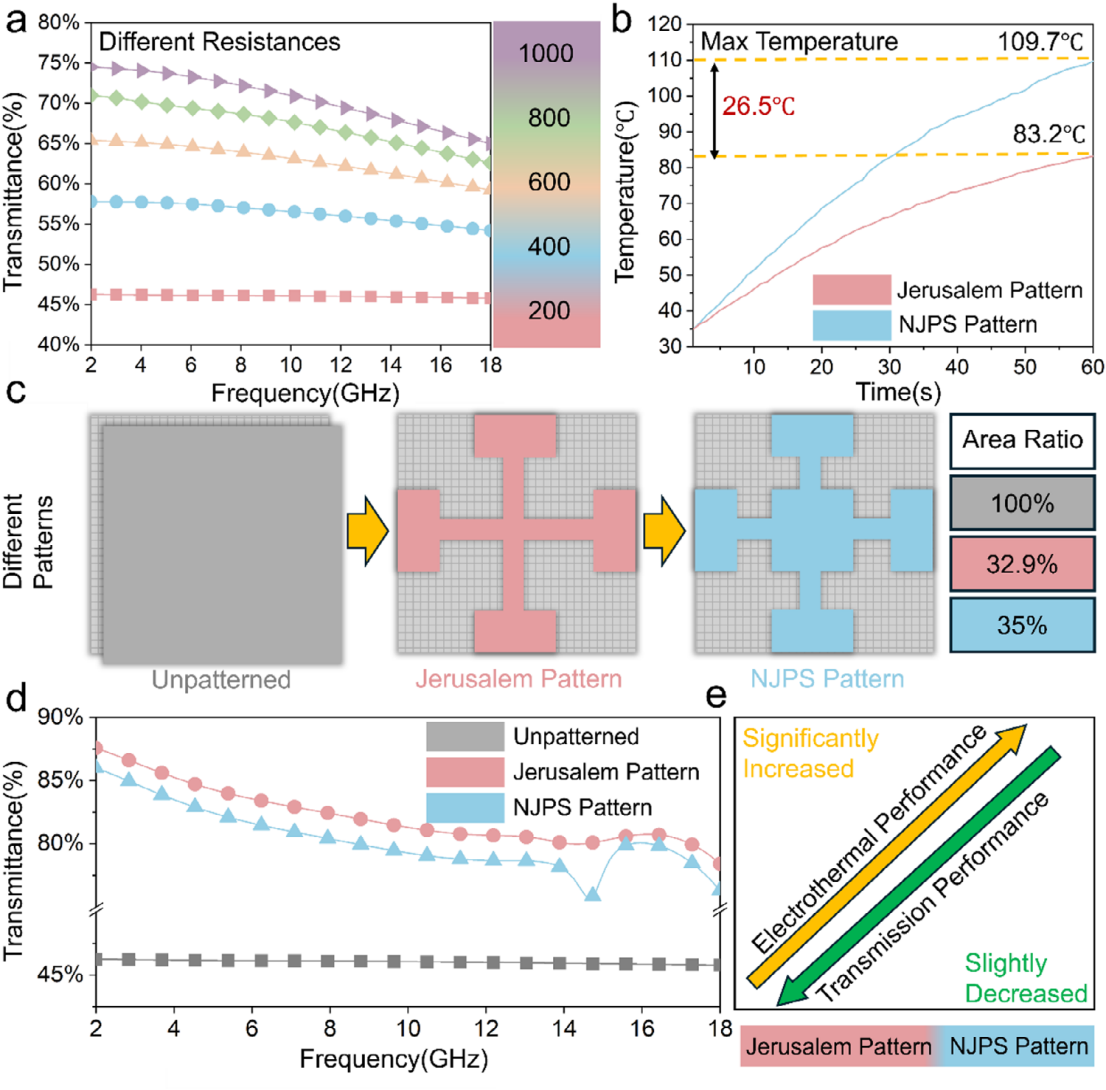
a) Wave‐transmission properties of full‐coverage films with different square resistances; b) Electrothermal properties of Jerusalem structure and NJPS at room temperature; c) Different patterned structures; d) Wave‐transmission properties of different patterned structures; e) Performance variations of the Jerusalem structure and NJPS.

Due to the potential for high electrical conductivity can reflect electromagnetic waves, the transmission performance of rGO/WPU electrothermal coatings within the radar frequency band (2–18 GHz) was studied. Simulations were done using CST Microwave Studio software to model how electromagnetic wave transmission through rGO/WPU electrothermal coatings with varying sheet resistances. The simulation unit cell included an rGO electrothermal coating layer and a polyimide (PI) substrate layer. The PI layer was 0.1 mm thick with a dielectric constant of 4. The electrothermal coating was modeled as a resistive sheet. The simulation results (**Figure** **4**a) show that a coating sheet resistance of 200 Ω/sq results in a transmission coefficient of only 46% across the band. As the sheet resistance increases, the electromagnetic wave transmission coefficient also increases. At a sheet resistance of 1000 Ω/sq, the transmission coefficient reaches 75%. However, even at this high sheet resistance, transmission does not meet the common goal of 80% for efficient transmission. Moreover, such a high sheet resistance indicates very low electrical conductivity, making the coating ineffective for electrothermal heating. This highlights the key challenge: a continuous, full coverage rGO electrothermal film cannot both provide efficient electrothermal heating and maintain high electromagnetic wave transparency.

Therefore, the Jerusalem structure was designed based on frequency‐selective surface (FSS) theory to simultaneously balance electrothermal efficiency and electromagnetic wave transmission. It is well‐established that electrothermal de‐icing operates by melting the ice layer at its interface with the substrate, thereby transforming the solid‐ice interface into a solid‐water‐ice interface, which facilitates ice shedding.^[^ ^45^, ^46^ ^]^ Localized heating can also spread, encouraging the detachment of larger ice sections. However, the traditional Jerusalem cross structure has a limited ability to concentrate thermal energy. As shown in Figure 4b, after 60 s of heating, the Jerusalem cross structure reaches a maximum steady‐state temperature of only 83.2 °C. To overcome this limitation, the Novel Jerusalem Pattern Structure (NJPS) was created, as shown in Figure 4c. Significantly, the NJPS attains a much higher maximum steady‐state temperature of 109.7 °C, a 26.5 °C increase over the conventional Jerusalem cross design. The electromagnetic transmission performance of the NJPS, the conventional Jerusalem structure, and the unpatterned structure was simulated and compared, as shown in Figure 4d. Both FSS structures (NJPS and Jerusalem structure) exhibit significantly higher transmission coefficients across the band compared to the unpatterned structure. While the NJPS provides substantially better electrothermal performance than the traditional Jerusalem cross, this improvement comes with a slight trade‐off in transmission performance. Notably, this enhanced heating is achieved with only a small increase in conductive area coverage, from 32.9% (Jerusalem cross) to 35.0% (NJPS). Therefore, the NJPS achieves a notable boost in heating capability with only a minimal reduction in transmission efficiency. Further optimization of the NJPS unit cell parameters will be conducted to enhance its transmission performance while maintaining high heating efficiency.

### 3.3 Optimization of NJPS Geometry Parameters

**Figure 5:**
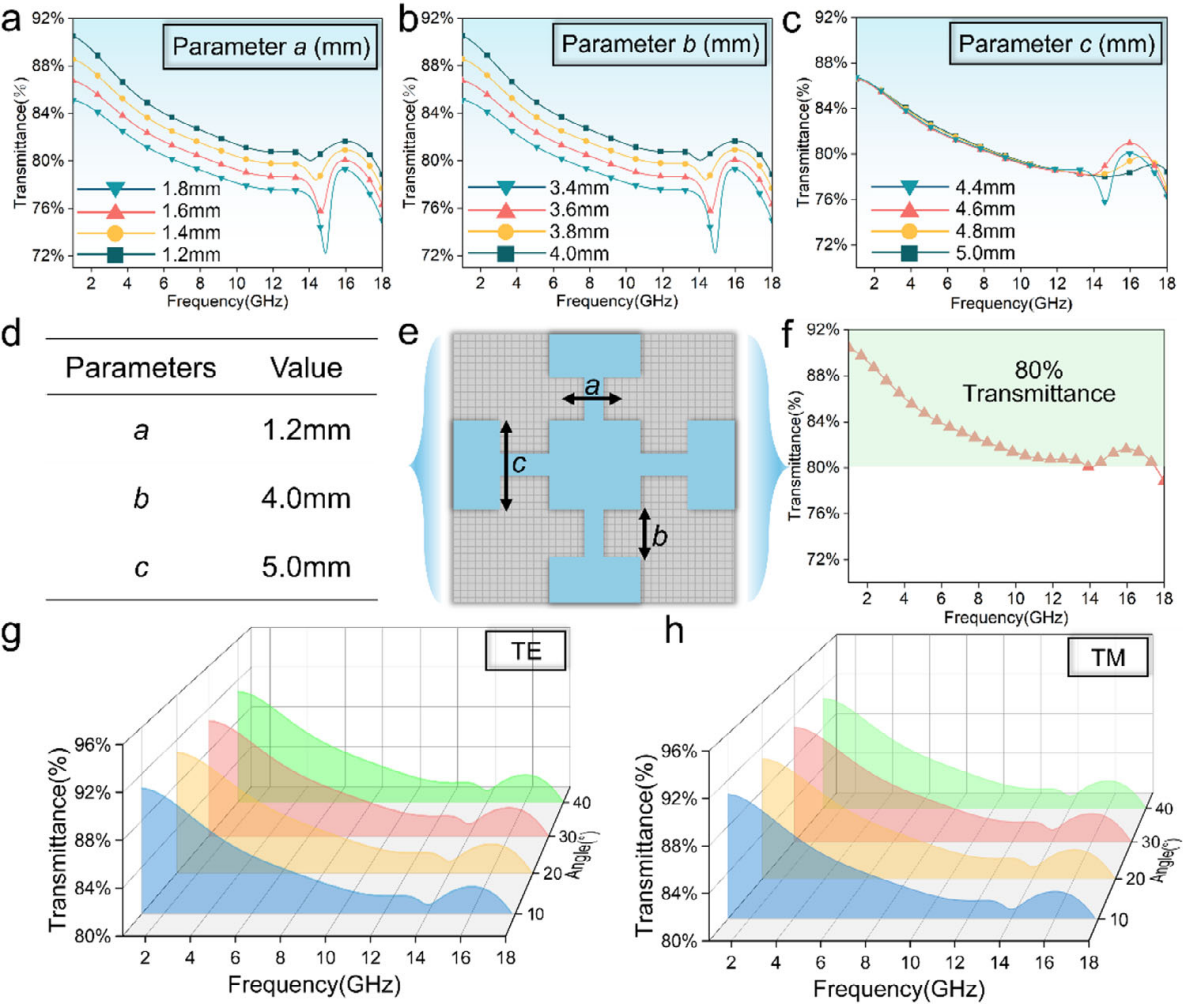
a–c) Optimization of dimension: *a*, *b*, *c*; d) The final parameters after parameter optimization; e) Feature size of NJPS; (f) Wave transmission performance under the optimal parameters; g,h) Wave transmission performance at different angles under TE and TM polarization.

To improve microwave transmittance without reducing electrothermal efficiency, the unit cell parameters (*a*, *b*, *c*) of the NJPS were optimized. Parameter sweeps were conducted using CST Microwave Studio simulations. For each sweep (such as parameter *a*), the other parameters (*b* and *c*) were kept constant. Four discrete values were tested for each parameter. The simulation results for the transmission coefficient across the 2–18 GHz band are shown in **Figure** **5**a–c.

Importantly, the transmission coefficient directly controls the electromagnetic wave transmittance of the structure. An increase in S21 indicates higher transmittance, meaning a larger portion of incident electromagnetic waves passes through the structure successfully. Conversely, a decrease in S21 signifies lower transmittance, reflecting more reflection or absorption losses. The relationship between the magnitude of the transmission coefficient and transmittance is described by the following Equations (4) and (5):

(4) $$S21\left(dB\right)=20\mathrm{lg}\left(S21\right)$$

(5) $$T=S21^{2}$$

Among the swept parameters (*a*, *b*, *c*), parameter *a* shows the most significant influence on the transmittance of the NJPS. This is because decreasing the width of the unit cell element results in a gradual increase in the transmission coefficient's magnitude. This occurs because reducing the width of the conductive film enhances electromagnetic resonance within the gap regions of the structure, thereby increasing the transmission coefficient. Simultaneously, a decrease in parameter* a* indicates that current flows in a smaller area, which usually raises the current density and could increase resistance. In contrast, parameter *c* has a negligible effect on the overall transmittance. Considering fabrication constraints (such as precision requirements) alongside the desired microwave transmittance performance, the following dimensions were chosen for the NJPS unit cell (see Figure 5d): *a*  = 1.2 mm; *b*  = 4.0 mm; *c*  = 5.0 mm (parameter *c* was set to 5.0 mm mainly to maintain electrothermal performance since it has minimal impact on transmittance). As shown in Figure 5f, the optimized NJPS achieves microwave transmittance above 80% across the entire 2–18 GHz radar band, with a peak transmittance of 88% within the 1–3 GHz band.

Since electromagnetic waves do not always strike a surface at normal incidence, and stability at wide angles is vital for transmission materials, the transmittance of the NJPS structure was systematically examined under both TM and TE polarizations at incident angles of 10°, 20°, 30°, and 40°, as shown in Figure 5g,h. For both polarizations, the transmittance exceeds 85% within the 2–7 GHz sub‐band across the entire evaluated oblique incidence range (10°–40°). Over the full 2–18 GHz radar band, the NJPS maintains a transmittance above 80% at all tested oblique angles (10°‐40°) for both polarizations. This outstanding performance demonstrates the strong angular stability of the NJPS structure. The symmetrical design ensures consistent transmission characteristics for both TM and TE polarizations under oblique incidence.^[^ ^47^ ^]^ These results confirm that the NJPS structure provides excellent oblique‐incidence transmission performance and maintains strong angular stability within the 10° to 40° range.

### 3.4 Validation of NJPS electrothermal and transmission performance

**Figure 6:**
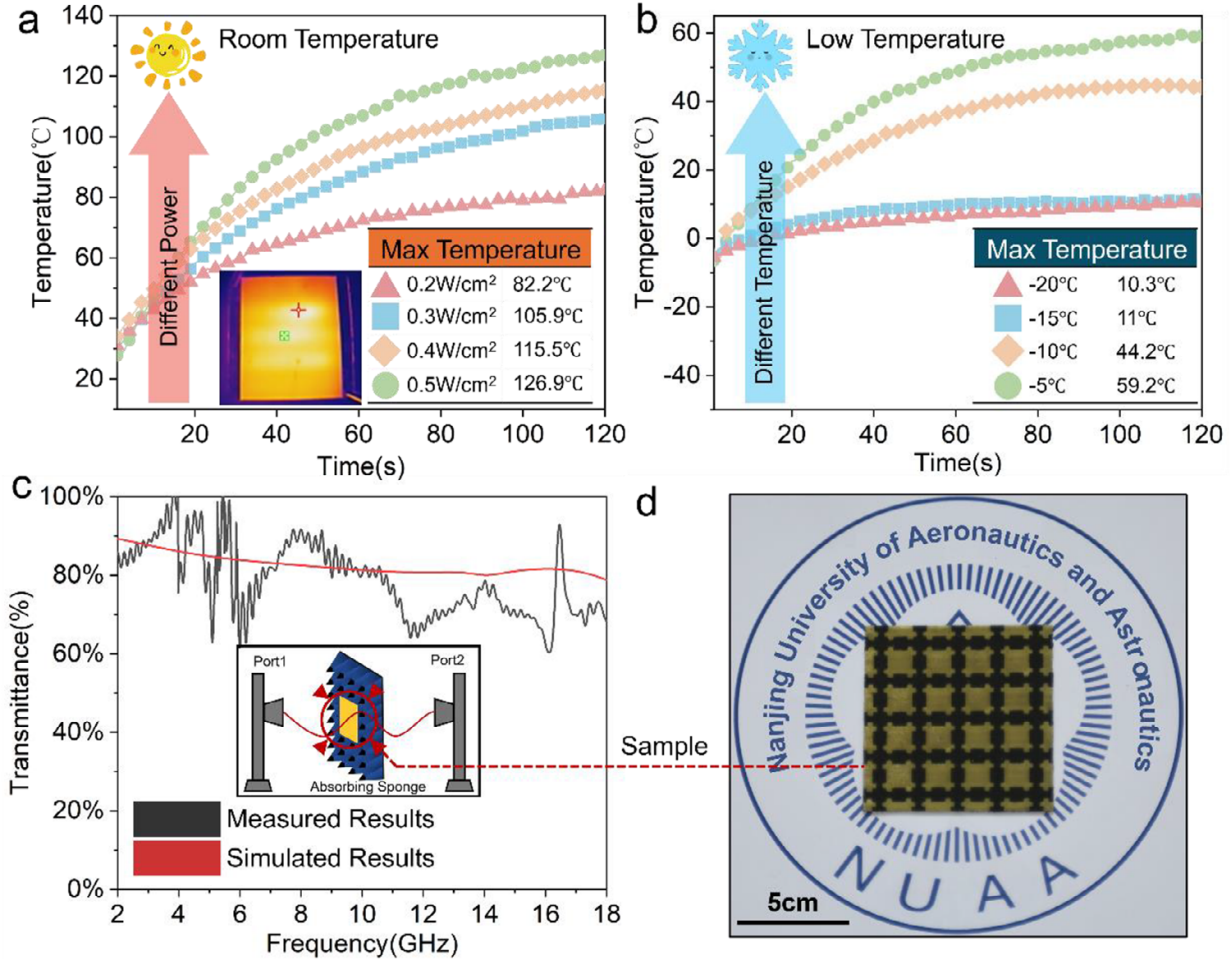
a) The electrothermal performance of NJPS with different powers at room temperature; b) The electrothermal performance of NJPS with different powers at low temperature; c) Wave transmission performance test; d) NJPS samples.

To assess the electrothermal performance of the NJPS at both room temperature and low temperatures, the temperature change curves of the NJPS were measured under these conditions. The results are shown in **Figure** **6**a,b. First, the actual input power density on the surface of the NJPS heating unit was calculated using the input power of the power supply and the surface area of the NJPS heating element. The temperature rise curves of the NJPS under different input power densities were tested at room temperature, demonstrating that, under the same input power density,^[^ ^48^ ^]^ the surface temperature of the coating increases, but the rate of temperature rise slows down, which can be explained by Equation (6). In the temperature growth region, the temperature increase over time can be expressed as:

(6) $$\frac{T-T0}{Tm-T0}=1-\exp\left(\frac{-t}{\tau g}\right)$$

where: *T_0_* and *T_m_* are the initial temperature and the maximum steady‐state temperature reached by energizing the electrothermal film, respectively. *T* is an arbitrary temperature at time *t*, and *τ_g_* is the growth time constant.

Simultaneously, it was observed that the electrothermal performance of the coating improves with increasing power density. When the input power density reaches 0.5W/cm^2^, the surface temperature of the NJPS can rise by ≈120°C within 120s. This exceptional electrothermal performance indicates that the NJPS holds broad application prospects in the field of anti/de‐icing. In addition, the infrared thermal image of the NJPS at room temperature was tested to provide a more intuitive understanding of its thermal behavior. As shown in the inset of Figure 6a, the surface temperature distribution across the entire structure is relatively uniform. This indicates that, despite the sparsity of the electrothermal network, the heating performance remains stable and efficient. The uniform distribution of thermal energy suggests that the design effectively minimizes local hot spots or cold zones, which is crucial for maintaining consistent de‐icing performance and long‐term reliability in practical applications. Subsequently, the temperature profiles of the NJPS were tested under different low‐temperature environments. It can be seen that as the ambient temperature decreases, the electrothermal performance of the NJPS gradually deteriorates. However, limited by the thermal conduction properties of the NJPS, during heating in low‐temperature environments, heat dissipation is accelerated, resulting in overall lower temperatures. Nevertheless, at an input power density of 0.5 W cm^−^^2^, the surface temperature of the NJPS still reaches 40 °C. This demonstrates that the NJPS possesses the capability for de‐icing at low temperatures. The thermal cycling stability of the NJPS is also verified; the results are shown in Figure S2 (Supporting Information).

To verify the transmittance performance of the NJPS within the 2–18 GHz band, an 18 cm × 18 cm sample was created using laser engraving based on the previously optimized parameters. The transmittance was measured using the free‐space method: the S‐parameters were recorded over the 2–18 GHz frequency range, and the transmittance (T) was calculated from these S‐parameters. The results are shown in Figure 6c, alongside a photo of the fabricated sample in Figure 6d. The NJPS sample achieves a transmittance of over 80% throughout the entire 2‐18 GHz band. Notably, within the 2–5 GHz sub‐band, the transmittance reaches up to 90%. There is excellent agreement between the measured transmittance and the simulation results. This confirms that the fabricated NJPS has effective broadband wave‐transparent capabilities. Minor differences and fluctuations between the measured and simulated curves are mainly due to slight variations in the sample dimensions compared to the design and measurement uncertainties in the testing environment.

**Figure 7:**
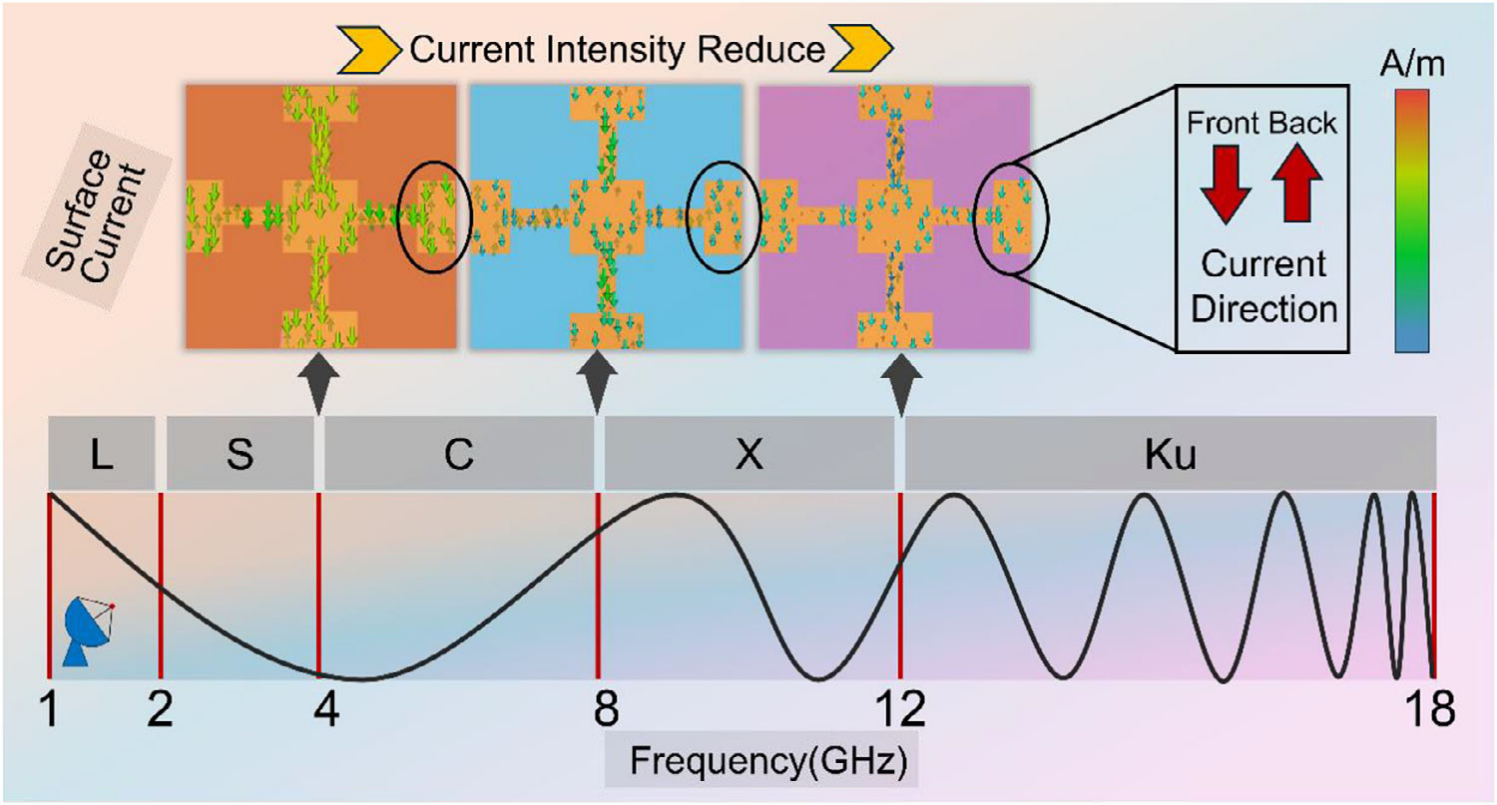
Surface current of NJPS at different Frequencies.

To clarify the wave‐transparent mechanism of the NJPS, the surface current distributions induced at different frequencies were simulated. The results are shown in **Figure** **7**. The transmission behavior of the NJPS under electromagnetic wave incidence can be mainly explained by the surface‐induced currents on the unit cell structure. These currents produce scattered fields, and the total transmission response results from the combination of the incident field and these scattered fields.^[^ ^49^ ^]^ Importantly, the scattering properties are naturally different for various unit cell geometries. Therefore, analyzing the surface‐induced current distribution on the NJPS unit cell under EM wave excitation provides important insights into its transmission behavior. Figure 7 illustrates the simulated surface current distribution on the NJPS unit cell under normal incident wave excitation. Two sets of induced currents primarily flow along the y‐direction on the opposite surfaces of the NJPS structure. However, these currents have antisymmetric flow directions, meaning they flow in opposite directions on the front and back surfaces. Because of this antisymmetric, the scattered fields produced by these opposing currents are directed in opposite directions. This causes significant mutual cancellation of the scattered fields, effectively reducing the overall scattered field magnitude. Such suppression of the total scattered field leads to decreased reflection and, as a result, increased transmission through the NJPS structure. When the incident wave frequency matches the resonant frequency of the NJPS unit cell, the transmission reaches its maximum, coinciding with the minimum reflection. At this resonance, the phase relationship between the suppressed total scattered field and the incident field creates a transmission peak.

The analysis also shows that the current intensity within the NJPS unit cell is generally lower in the low‐frequency range compared to the high‐frequency range. This suggests that more electromagnetic energy is lost (e.g., through ohmic losses or localized heating) within the NJPS structure at higher frequencies. This finding consistently aligns with the measured better transmittance performance of the NJPS in the low‐frequency range. Supporting the current analysis, the transmission can also be explained from an electron oscillation perspective. When the electromagnetic wave hits the structure normally and its electric field polarization is perpendicular to the direction of the conductive strips in the NJPS pattern, the electrons inside the structure experience minimal force from the electric field component along the strips. As a result, little electron oscillation occurs, allowing the electromagnetic wave to pass through the NJPS with minimal interaction and high transmittance.

### 3.5 De‐Icing Performance of NJPS Under Incoming Flow Conditions

**Figure 8:**
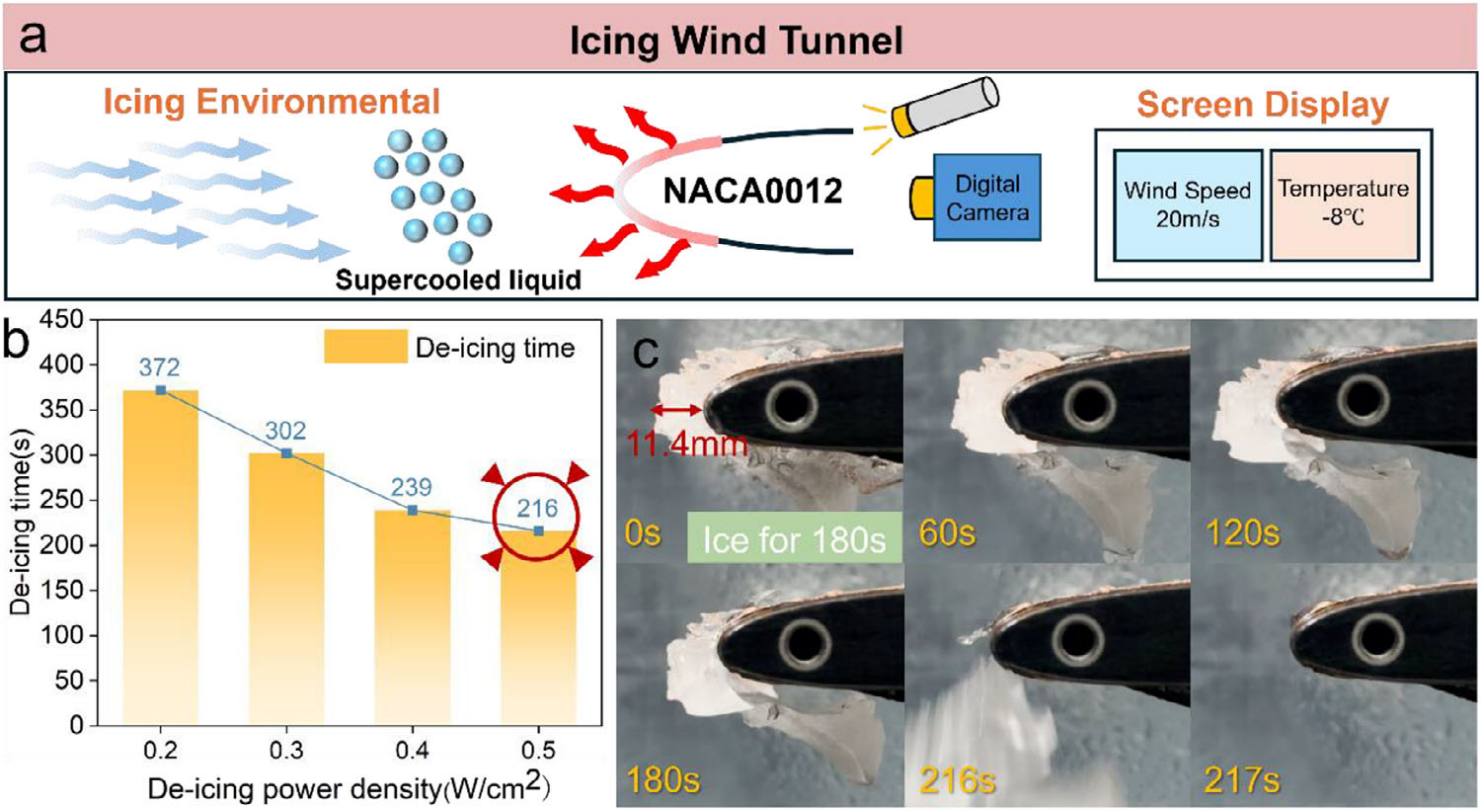
a) Diagram of the experimental setup of the icing wind tunnel; b) De‐icing time of NJPS at different power densities; c) De‐icing process at 0.5W cm^−2.^

Ideally, keeping an aircraft's surface free of ice while flying through supercooled cloud layers offers the best protection for its aerodynamics. However, the anti‐icing process needs a continuous power supply to the electrothermal coating, which requires very high energy consumption. As a result, within the aircraft's performance limits, it is often acceptable to allow some ice to form on the surface before using the Joule heating effect of the electrothermal coating to remove it. This study selected a typical cryogenic incoming flow condition for testing: an ambient temperature of ‐8 °C and an airspeed of 20 m/s. A schematic diagram of the experimental setup in the icing wind tunnel is shown in **Figure** **8**a, and the size of the supercooled droplets simulated in the tunnel is shown in Figure S3 (Supporting Information). To ensure ice growth only on the surface of the electrothermal coating, independent heating modules installed at both ends of the airfoil model were activated, preventing ice from forming on the wingtips. Afterward, ice was allowed to build up on the central electrothermal coating section for 180 s under simulated cloud conditions. Then, the spray system in the icing wind tunnel was turned off. Different input power densities were applied to the NJPS specimen, and its de‐icing performance was tested under each level. Effective de‐icing was defined as completely removing the ice layer within 300 s.

Firstly, the de‐icing time and the maximum steady‐state temperature of NJPS were tested under different powers, as shown in Figure 8b. As input power increases, the de‐icing time gradually decreases. When the input power is 0.2 W cm^−^^2^, the de‐icing time is 372 s, and the maximum surface temperature of the NJPS at this power is only 5.9°C. Although this exceeds the melting point of ice, it cannot be achieved quickly. With continuous increases in input power, de‐icing can be completed within 300 s (the de‐icing time at 0.4 W cm^−^^2^ is 302 s, which is considered effective de‐icing), and the maximum steady‐state temperature also rises with higher input power. Next, the ice removal behavior of the NJPS surface was analyzed during the process at an input power of 0.5 W cm^−^^2^. Figure 8c illustrates the ice removal process. After 180 s, the ice layer thickness is 11.4 mm. The icing primarily occurs at the leading edge of the wing, and the ice layer appears white and porcelain‐like, with poor transparency. The leading edge shows a rugged appearance with bright and frosty ice, indicating a mixed ice formation. At 180 s of power‐on, part of the ice layer on the surface of the airfoil has melted. Although the surface temperature has reached the melting point of ice, it is not high enough to melt all the ice. The remaining parts remain firmly attached to the surface of the NJPS, which has a high adhesion force. Meanwhile, the wind force in the wind tunnel exerts pressure on the ice layer, pressing it firmly against the airfoil surface. The ice layer completely falls off at 216 s as heating continues to increase. The shedding of the ice is due to surface heating of the NJPS, causing nearly all the ice at the interface to melt. Additionally, a water film forms at the interface between the ice and the coating, reducing adhesion and acting as a lubricant for the entire ice layer. Ultimately, the ice detaches due to the shear force from the external wind and gravity. The combined effects of aerodynamic shear and gravity cause the ice to detach, resulting in effective de‐icing.

## 4 Conclusion 

In this work, a novel Jerusalem cross‐inspired frequency‐selective surface (FSS) was designed and fabricated to address the simultaneous requirements of electrothermal heating and electromagnetic transparency. GO was initially synthesized via Hummers’ method, followed by thermal reduction at 800°C to obtain rGO with an optimized reduction degree. The rGO served as a conductive filler in spray‐coated rGO/WPU electrothermal coatings, which demonstrated superior electrical conductivity and Joule heating performance. To resolve the inherent conflict between heating efficiency and wave transmission, a New Jerusalem Pattern Structure (NJPS) was engineered and benchmarked against unpatterned rGO/WPU coatings. The NJPS achieved dual functionality at just 35% conductive area coverage: effective localized heating (109.7 °C at 0.5 W cm^−^^2^) and high microwave transmittance. Parametric optimization established ideal geometric dimensions, ensuring angular stability up to 40° incidence. Laser‐engraved NJPS prototypes were validated under both ambient and cryogenic conditions, confirming exceptional electrothermal response and consistent broadband transmission. Crucially, icing wind tunnel tests verified rapid de‐icing capability (216 s at 0.5 W cm^−^^2^ under −8 °C/20 m/s flow) through interfacial water‐film lubrication. This work establishes a co‐design methodology for next‐generation aircraft that require integrated electrothermal de‐icing and radar‐transparent functionality.

## Conflict of Interest

The authors declare no conflict of interest.

## Author's Contribution

X.F. contributed to writing—review and editing, writing—original draft, visualization, validation, methodology, investigation, formal analysis, and conceptualization. Y.S. contributed to writing—review and editing, writing—original draft, methodology, investigation, conceptualization, and funding acquisition. L.Z. contributed to visualization, validation, and investigation. W.Z. contributed to validation, project administration, methodology, investigation, formal analysis, and conceptualization. W.L. contributed to writing—review and editing, writing—original draft, visualization, validation, project administration, methodology, investigation, formal analysis, and conceptualization. C.L. contributed to validation, project administration, methodology, and investigation. Y.L. contributed to methodology and investigation. Z.Y. contributed to methodology. C.S. contributed to investigation, formal analysis, and conceptualization.

## Supporting information



Supporting Information

## Data Availability

Research data are not shared.

## References

[1] Y. Cao , W. Tan , Z. Wu , Aerosp. Sci. Techn. 2018, 75, 353.

[2] N. Dalili , A. Edrisy , R. Carriveau , Renew. Sustain. Energy Rev. 2009, 13, 428.

[3] A. Kraj , E. Bibeau , Renew. Energy 2010, 35, 966.

[4] H. Ducloux , B. Nygaard , Cold Reg. Sci. Technol. 2018, 153, 120.

[5] D. I. Jeong , L. Sushama , M. J. F. Vieira , K. A. Koenig , Sustain. Cities Soc. 2018, 39, 639.

[6] M. Politovich , J. Appl. Met. Clim. 1989, 28, 856.

[7] F. Lynch , A. Khodadoust , Progr. Aerosp. Sci. 2001, 37, 669.

[8] J. Jiang , Y. Shen , Y. Xu , Z. Wang , J. Tao , S. Liu , W. Liu , H. Chen , Nat. Commun. 2024, 15, 777. 38278811 10.1038/s41467-024-45078-5 PMC10817900

[9] Z. Wang , Y. Shen , S. Liu , J. Jiang , Y. Xu , W. Liu , J. Tao , Int. J. Therm. Sci. 2024, 205, 109301.

[10] X. Zhou , Y. Shen , Z. Wang , J. Jiang , S. Liu , W. Liu , Y. Lin , Appl. Therm. Eng. 2025, 266, 125704.

[11] Z. Fei , X. Jiang , Q. Zhao , Z. Cui , Y. Yang , Chin. J. Aeronaut. 2023, 36, 204.

[12] Y. Zhu , X. Guan , Z. Yang , X. Xu , J. Alloys Compd. 2021, 865, 158886.

[13] F. Qin , C. Brosseau , J. Appl. Phys. 2012, 111, 061301.

[14] F. Zhang , N. Li , J.‐F. Shi , L. Xu , L.‐C. Jia , Y.‐Y. Wang , D.‐X. Yan , Compos. Part B, Eng. 2024, 283, 111646.

[15] Y.‐Y. Wang , Z.‐H. Zhou , J.‐L. Zhu , W.‐J. Sun , D.‐X. Yan , K. Dai , Z.‐M. Li , Compos. Part B, Eng. 2021, 220, 108985.

[16] Z. Zhou , K. Chen , J. Zhao , P. Chen , T. Jiang , B. Zhu , Y. Feng , Y. Li , Opt. Express 2017, 25, 30241. 29221055 10.1364/OE.25.030241

[17] Z. Zhou , K. Chen , B. Zhu , J. Zhao , Y. Feng , Y. Li , IEEE Access 2018, 6, 26843.

[18] W. Li , M. Xu , H.‐X Xu , X. Wang , W. Huang , Adv. Mater. 2022, 34, 2202509. 10.1002/adma.202202509 35604541

[19] Y. Liu , Z. Xiang , Chem. Soc. Rev. 2011, 40, 2494.21234491

[20] Y. Xu , F. Wang , Y. Zhang , T. Shu , W. Huang , X. Yan , Y. Lei , S. Liu , X. Chen , Colloids Surf. A, Physicochem. Eng. Aspects 2025, 716, 136699.

[21] W. Zhang , X. Liu , C. Zheng , B. Zhang , Colloids Surf. A, Physicochem. Eng. Aspects 2025, 713, 136504.

[22] R. Ouyang , X. Li , X. Du , J. Alloys Compd. 2025, 1022, 180104.

[23] L. Wang , M. Liu , Y. Wu , H. Zheng , Chem. Eng. J. 2024, 488, 150862.

[24] L. Jiang , J. Sun , Y. Lin , M. Gong , K. Tu , Y. Chen , T. Xiao , P. Xiang , X. Tan , Surf. Coat. Technol. 2024, 476, 130273.

[25] P. Xu , D. Zhang , W. Gao , A. Li , Appl. Acoust. 2024, 221, 110034.

[26] H. Habibi , L. Cheng , H. Zheng , V. Kappatos , C. Selcuk , T.‐H. Gan , Renew. Energy 2015, 83, 859.

[27] B. G. Falzon , P. Robinson , S. Frenz , B. Gilbert , Compos. Part A, Appl. Sci. Manuf. 2015, 68, 323.

[28] J. Ji , P. Huang , The Principle of Stealth, Beijing University of Aeronautics and Astronautics Press, Beijing, 2020, 35

[29] J. Chen , Z. Zhao , Y. Zhu , Y. Xu , L. Yuan , L. Zhang , Z. Wang , X. Liu , H. Chen , Prog. Org. Coat. 2023, 183, 107751.

[30] V. Volman , Y. Zhu , A.‐R. O. Raji , B. Genorio , W. Lu , C. Xiang , C. Kittrell , J. M. Tour , ACS Appl. Mater. Interfaces 2013, 6, 298. 24328320 10.1021/am404203y

[31] A. R O. Raji , S. Salters , E. L. G. Samuel , Y. Zhu , V. Volman , J M. Tour , ACS Appl. Mater. Interfaces 2014, 6, 16661. 25188912 10.1021/am503478w

[32] J.‐W Hong , J. H. Jung , S.‐M. Yong , Y.‐R. Kim , J. Park , S. J. Lee , J.‐H. Choi , J. Mater. Res. Technol. 2020, 9, 10854.

[33] R. Anwar , L. Mao , H. Ning , Appl. Sci. 2018, 8, 1689.

[34] B. Lin , W. Huang , J. Guo , X. Ji , Y. Zhou , Y. Wu , Opt. Commun. 2023, 530, 129202.

[35] S. K. Patel , V. Sorathiya , S. Lavadiya , T. K. Nguyen , V. Dhasarathan , Phys. E: Low‐Dimens. Syst. Nanostructures 2020, 120, 114049.

[36] C. Ma , B. Xiao , D. Zhou , L. Xiao , Opt. Commun. 2021, 478, 126375.

[37] G. Zhou , Z. Zhao , Y. Zhang , W. Liu , Z. Yang , D. Jia , Y. Zhou , J. Mater. Sci. Technol. 2022, 111, 49.

[38] H. Hwang , K. Y. Ma , J. W. Kim , D. Yuk , J. Hong , J. H. Jung , S.‐M. Yong , J. Choi , J. Y. Kim , H. S. Shin , Nanoscale 2020, 12, 21895. 33107899 10.1039/d0nr06333a

[39] Y. Yang , B. Wu , H. Li , S. Sun , X. Liu , L. Chen , presented at Cross Strait Quad‐Regional Radio Science and Wireless Technology Conf. (CSQRWC) , Taiyuan, China, July 2019.

[40] J. Chen , Y. Bai , Z. Zhao , Y. Zhu , Z. Wang , S. Sun , Y. Xu , L. Yuan , L. Zhang , X. Liu , H. Chen , Mater. Des. 2024, 246, 113.

[41] Z. Xia , F. Liu , X. Tang , X. Cao , Q. Cai , IEICE Electronics Express 2019, 16, 1.

[42] B. Lin , W. Huang , J. Guo , Opt. Commun. 2023, 530, 129.

[43] W. Liu , L. Zhao , Y. Shen , Z. Zhang , Y. Ni , X. Fu , Y. Lin , L. Chen , C. Shen , Surf. Interfaces 2025, 59, 105969.

[44] Z. Wang , Y. Shen , J. Tao , S. Liu , J. Kiang , Y. Xu , W. Liu , Heat Transfer Res. 2025, 56, 1.

[45] L. Zhao , Y. Shen , W. Liu , J. Tao , S. Liu , Surf. Interfaces 2023, 42, 103.

[46] Z. Wang , Y. Shen , S. Liu , J. Jiang , Y. Xu , W. Liu , J. Tao , Int. J. Therm. Sci. 2024, 205, 109.

[47] S. Das , A. Rajput , B. Mukherjee , IEEE Letters on Electromagnetic Compatibility Practice and Applications, IEEE, New York 2023.

[48] L. Vertuccio , F. Santis , R. Pantani , Compos. Part B, Eng. 2019, 162, 600.

[49] J. Zhou , S. Bie , D. Wan , IEEE AntennasWirel. Propag. Lett. 2014, 14, 24.

