# Supplementary material for: Laser‐Engraved Micro‐Patterned rGO/WPU Composite Structures for Aircraft Applications: Synchronizing Electrothermal De‐Icing and Broadband Microwave Transparency
Source: Adv Sci (Weinh). 2025 Sep 8;12(45):e12521. doi: 10.1002/advs.202512521 (PMC12677629; doi:10.1002/advs.202512521)
Supplement: Supplementary file 1 — Supporting Information [file ADVS-12-e12521-s001.docx]

**Supporting Information**

Laser-Engraved Micro-Patterned rGO/WPU Composite Structures for Aircraft Applications: Synchronizing Electrothermal De-icing and Broadband Microwave Transparency

**Xu Fu^a,b,^, Yizhou Shen^a,b,^**^[[1]](#footnote-1)^***, Lingfeng Zhao^a,b,^, Weixin Zhu^a,b,^, Weilan Liu^c^, Chenliang Li^a,e,^, Yuebin Lin^d^, Zongsheng Ye^a,b,^, Chengfeng Shen^a,b,^**

*^a^ College of Materials Science and Technology, Nanjing University of Aeronautics and Astronautics, Nanjing 211100, China*

*^b^ State Key Laboratory of Mechanics and Control for Aerospace Structures, Nanjing University of Aeronautics and Astronautics, Nanjing 210016, China*

*^c^ Institute of Advanced Materials, Nanjing Tech University, 30 Puzhu South Rd., Nanjing 210009, P. R. China*

*^d^ Faculty of Mechanical and Material Engineering, Huaiyin Institute of Technology, Huai’an 223003, China*

*^e^ZhongXing Energy Enquipment Co,Ltd., Nantong 226100, China*

**Preparation and characterization of rGO**

GO was prepared using the modified Hummer’s method, in which flake graphite was added to a mixture of H_2_SO_4_, H_3_PO_4_, and KMnO_4_. The mixture reacted in an ice-water bath for 2 hours, then was warmed to 50 ℃ and continued to react with stirring for 3 hours. The viscous liquid from the reaction was poured into an ice-water mixture, with temperature carefully controlled. H_2_O_2_ was added to the system under stirring at a concentration of 30%. The resulting product was centrifuged and washed with 5% HCl, with centrifugation repeated until the pH of the solution reached 6–7. The solution was then ultrasonically dispersed and freeze-dried to obtain the powder GO. The GO powder was placed in a muffle furnace and heated to 800 ℃ at a rate of 5 ℃/min, then held for 1 hour. After cooling naturally to room temperature, the process was carried out under high purity argon gas flow, with an inlet rate of 3 L/min, resulting in the powder. rGO was obtained similarly.

Figure S1a shows the XRD patterns of GO and rGO samples within the 2θ range of 5° to 90°. The GO pattern displays a sharp peak at 11.4°, representing the typical (001) diffraction peak of GO. The interlayer spacing of GO, calculated using Bragg's law (nλ=2d sin θ, where λ=1.5406 Å and n=1), is approximately 0.776 nm. This increased spacing results from oxygen-containing functional groups incorporated during high-temperature oxidation. After reduction at 800 °C, the rGO pattern shows a broad diffraction peak at about 26.2°, and the GO (001) diffraction peak at 10.2° disappears. This change is due to the removal of oxygen groups under high heat. However, the reduced graphene oxide sheets display poor stacking order and increased disorder. As a result, the crystalline structure of RGO is less ordered than that of pristine graphite, leading to a broad, diffused peak around 25.3°.

Figure S1b shows the Raman spectra of GO and rGO. The D band (1353 cm⁻¹) results from sp3 carbon atoms or lattice defects, and its intensity reflects the material's degree of disorder. The G band (1592 cm⁻¹) comes from the in-plane stretching vibrations of sp2 carbon atoms. The ratio of D to G band intensities (ID/IG) is often used to assess the structural quality within the carbon layers; a higher ID/IG ratio indicates less regular structure and more disorder. As seen in the Raman spectra, the rGO shows increased D-band intensity and decreased G-band intensity after high-temperature treatment (800 °C). As a result, the ID/IG ratio rises from 0.96 to 1.27. This suggests a greater presence of lattice defects and SP3 hybridized carbon atoms in the rGO, due to increased disorder from the removal of oxygen-containing functional groups. These results align with the XRD results.

Figure S1c displays the XPS survey spectra of GO and rGO. Both spectra show distinct C1s and O1s peaks. Compared to GO, rGO shows an increased intensity of the C1s peak and a decreased intensity of the O1s peak, indicating a reduction in oxygen content and an increase in carbon content. The deconvoluted C1s spectra of GO and rGO are shown in Figure S1d and Figure S1e, respectively. The deconvolution reveals a notable increase in the peak intensity associated with C-C/C=C bonds (284.8 eV) in rGO. Conversely, the peaks linked to oxygen-containing functional groups, such as C-O (287.0 eV), C=O (288.0 eV), and O-C=O (289.2 eV), are significantly reduced. This indicates an increase in C-C/C=C bonds and a marked decrease in oxygen-containing groups after reduction. However, a small amount of residual oxygen-containing groups remains. This residual oxygen may result from functional groups trapped within the rGO sheets, preventing their complete removal during the high-temperature reduction process. Additionally, the atomic C/O ratio rises sharply to 6.09. This higher ratio signifies that the rGO has achieved a high degree of graphitization. Figure S1f summarizes the main changes in rGO compared to GO after high-temperature reduction, confirming the excellent degree of reduction of rGO.


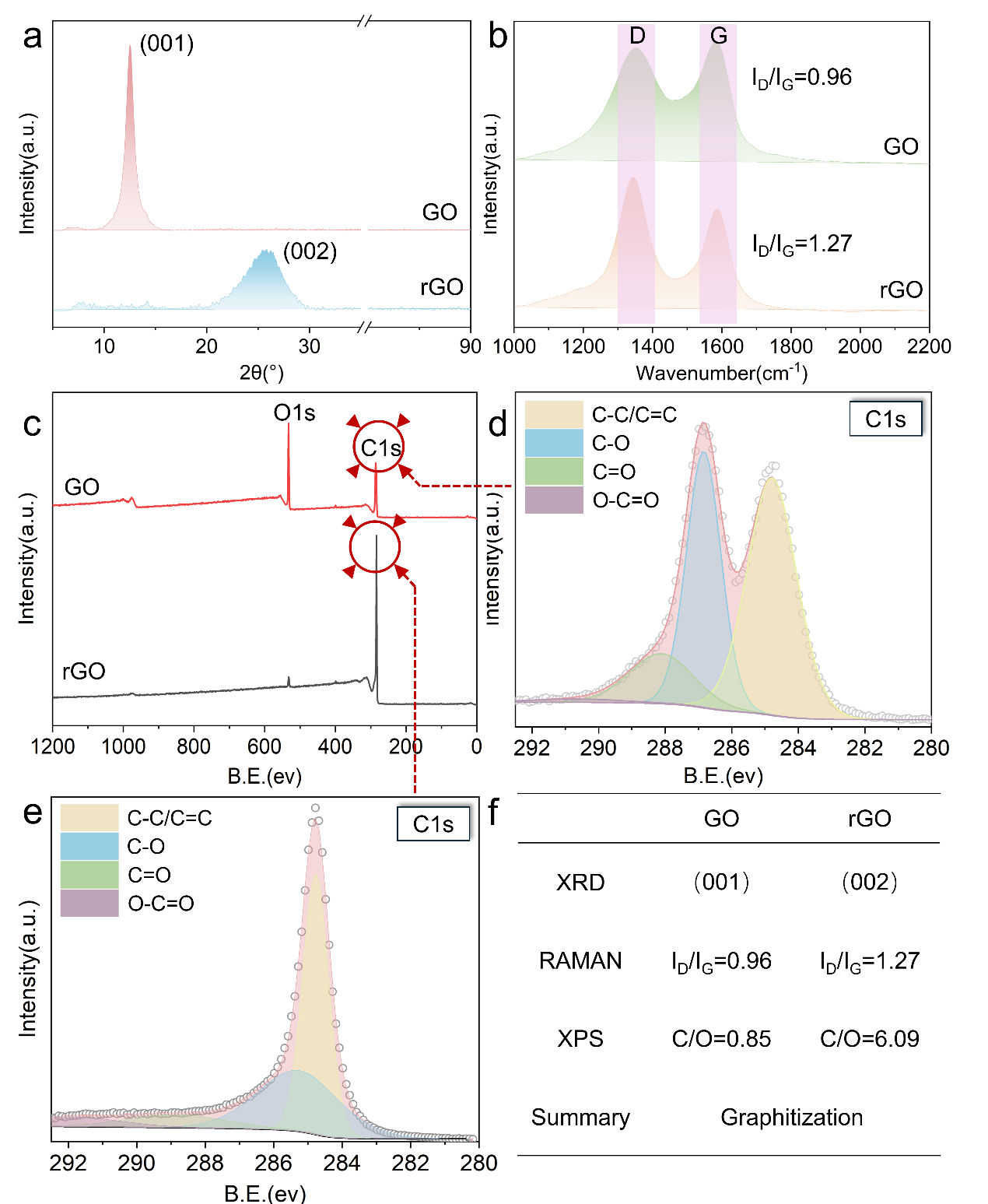


**Figure. S1** (a) XRD results of rGO and GO; (b) RAMAN results of rGO and GO; (c) XPS results of rGO and GO; (d) C1s of GO; (e) C1S of rGO; (f) rGO relative to the degree of reduction with GO

**The thermal cycling and deicing stability of NJPS**

To verify the cyclic heating capability of the coating, we conducted 50 repeated cycles of electrical heating and natural cooling tests. The temperature rise curve was recorded every 9 cycles. Each cycle consisted of 300 seconds of powered heating followed by 200 seconds of cooling, simulating the periodic on-off load characteristics in real-world operating conditions. The test results are shown in Figure S2a. As observed, the coating exhibits a rapid temperature response during each cycle. The surface temperature increases quickly during the heating phase and naturally cools back to the initial state once the power is turned off. Throughout all 50 cycles, the peak temperature of the film remained consistent, without significant decrease or drift, indicating that the coating maintains stable heat transfer capability even after repeated heating. Moreover, the temperature rise rate curves for each cycle nearly overlap, with no noticeable attenuation or delay, further confirming the thermal conductivity stability of the CNT/WPU film under thermal load cycling.

To further evaluate the structural integrity of the NJPS under actual service conditions, we conducted 10 icing–de-icing cycles following the testing conditions described in the manuscript, i.e., wind speed of 20 m/s, ambient temperature of –8 °C, and an icing duration of 180 s. During the tests, the ice thickness and de-icing time for each cycle were recorded, and the results are presented in Figure S2b. As shown, the ice thickness remained nearly consistent across all 10 icing cycles. Likewise, the de-icing time exhibited minimal variation, with an average of 212.2 s, which is close to the 216 s reported in the main manuscript. These results confirm the stability of the NJPS during repeated icing–deicing cycles. Additionally, no structural damage was observed throughout the test, further validating the structural integrity of the NJPS.


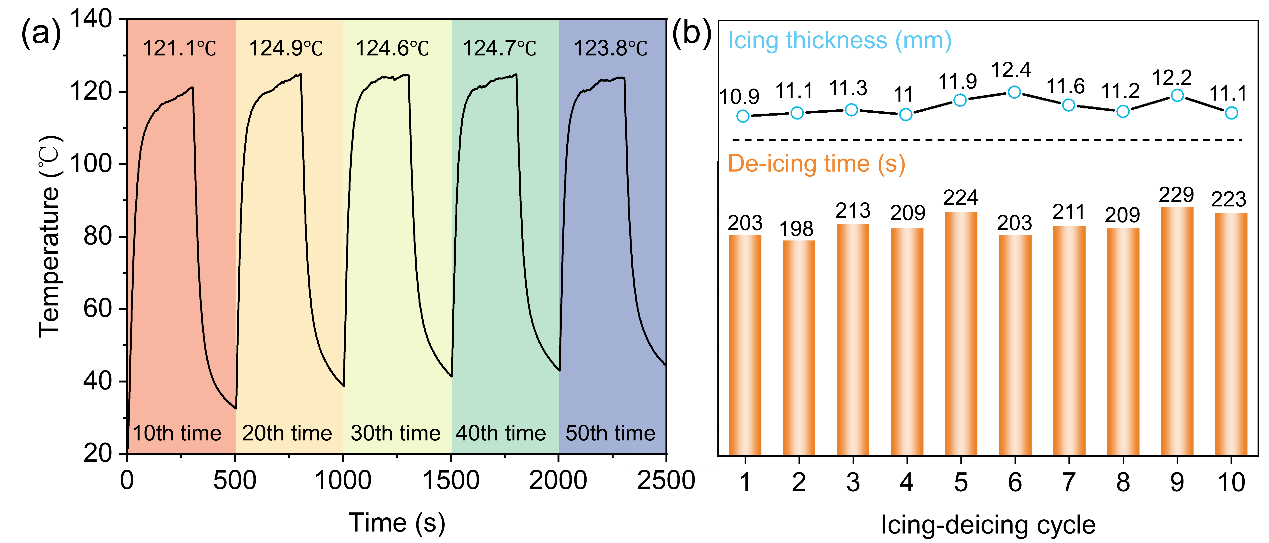


**Figure. S2** (a) The electrothermal performance of NJPS after 50 heating cycles; (b) Ice thickness and de-icing time during 10 icing–de-icing cycles

**Droplet size in an icy wind tunnel**

In this study, we used a laboratory-made small-scale icing wind tunnel to simulate real-world airflow conditions. High-speed cameras were employed to capture images of droplet movement at -8°C, from which we performed size distribution analysis. By controlling the nozzle air pressure and water pressure at 0.2 MPa, we adjusted the average droplet volume diameter to range between 15 μm and 25 μm, matching the typical droplet size of supercooled water droplets in clouds. The test results are shown in Figure S3. As observed, the average droplet size was approximately 22 μm, indicating that the internal conditions of our laboratory-made small-scale icing wind tunnel closely resemble those of real icing environments. Subsequently, the wind speed was controlled at 20 m/s, and the temperature was set to -8°C to ensure ice formation. By controlling the icing time, we were able to adjust the thickness of the ice layer, which reached approximately 11 mm after 180 s.


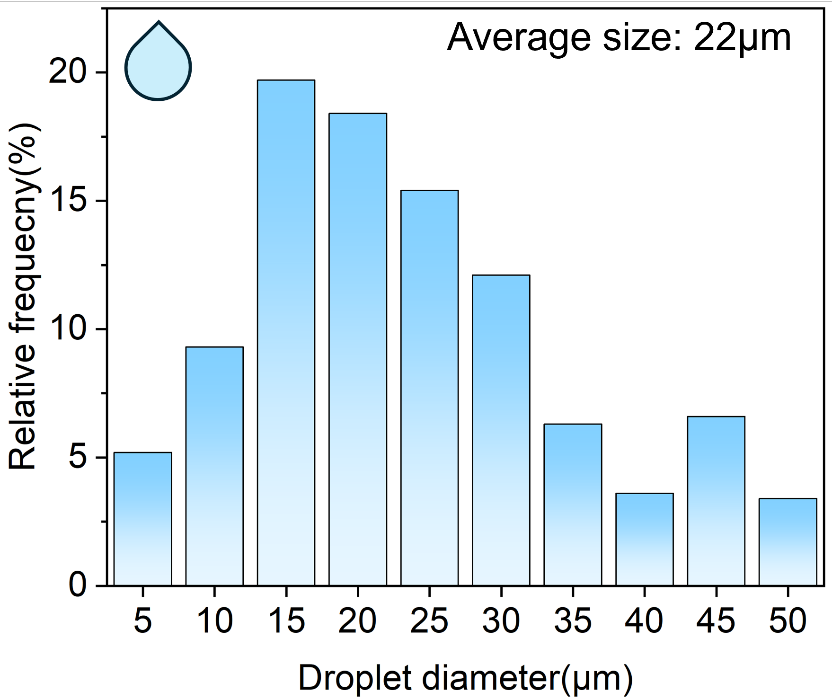


**Figure. S3** The size of the supercooled droplets in the freezing wind tunnel

1. * *Corresponding author： E-mail address:* [*shenyizhou@nuaa.edu.cn*](mailto:shenyizhou@nuaa.edu.cn) [↑](#footnote-ref-1)
